# ABC Cardiol de 2018–2025: Identidade, Impacto e Construção Coletiva

**DOI:** 10.36660/abc.20260582

**Published:** 2026-09-21

**Authors:** Carlos Eduardo Rochitte

**Affiliations:** 1 Hospital das Clínicas Faculdade de Medicina Universidade de São Paulo São Paulo SP Brasil Instituto do Coração do Hospital das Clínicas da Faculdade de Medicina da Universidade de São Paulo, São Paulo, SP – Brasil; 2 Hcor Associação Beneficente Síria São Paulo SP Brasil Hospital do Coração (Hcor), Associação Beneficente Síria, São Paulo, SP – Brasil; 3 Alta/DASA Medicina Diagnóstica São Paulo SP Brasil Alta/DASA Medicina Diagnóstica, São Paulo, SP – Brasil; 4 Blume Medicina Diagnóstica São Paulo SP Brasil Blume Medicina Diagnóstica, São Paulo, SP – Brasil

**Keywords:** Publicação Periódica, Fator de Impacto de Revistas, Publicações Científicas e Técnicas, Indicadores de Produção Científica

O período iniciado em 2018 representou uma nova etapa na trajetória editorial dos *Arquivos Brasileiros de Cardiologia*. Naquele momento, dois pilares foram estabelecidos como prioridades para o desenvolvimento da revista: a ampliação de sua internacionalização e o aumento de seu impacto científico.^1^ O fator de impacto permanecia pouco acima de 1, e a divulgação do índice de 1,318 foi então celebrada como uma conquista importante para a Sociedade Brasileira de Cardiologia e para toda a comunidade científica cardiovascular.^2^ Mais do que alcançar metas numéricas, buscava-se ampliar a capacidade da revista de divulgar a ciência cardiovascular produzida no Brasil e torná-la cada vez mais visível, relevante e acessível internacionalmente.

Uma das mudanças simbólicas daquele período foi a consolidação do uso do nome ABC Cardiol. O nome oficial e histórico, *Arquivos Brasileiros de Cardiologia*, foi integralmente preservado, mas passamos a apresentar a revista também por uma denominação mais curta, facilmente reconhecível e capaz de comunicar sua identidade no ambiente científico internacional. Com o tempo, ABC Cardiol deixou de ser apenas uma abreviação e se tornou uma marca editorial amplamente incorporada por autores, leitores, revisores e pela própria Sociedade Brasileira de Cardiologia.

Os números registram parte dessa trajetória. O fator de impacto passou de 1,318, referente a 2017, para 1,679 no ano seguinte e alcançou 2,0 em 2020.^2-4^ Posteriormente, atingiu o recorde histórico de 2,667, mais que o dobro do ponto de partida de nossa gestão. Após uma redução transitória para 1,9, o índice mais recente retornou a 2,3 (Figura 1). Essa evolução, naturalmente sujeita às oscilações próprias dos indicadores bibliométricos, demonstra a consolidação da revista em um novo patamar de visibilidade e, sobretudo, a capacidade de recuperação e continuidade de sua influência científica.

**Figura 1:**
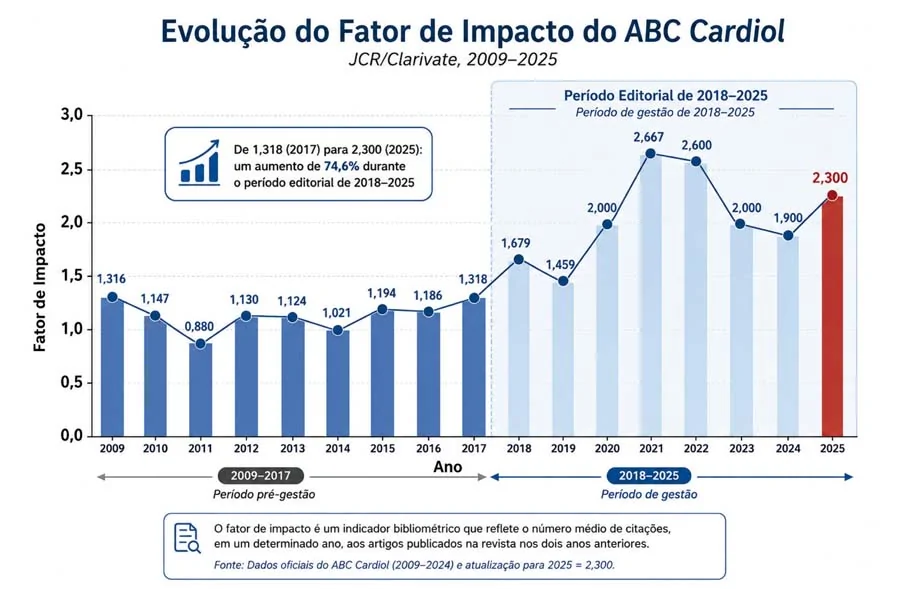
– Fator de impacto dos Arquivos Brasileiros de Cardiologia - Evolução temporal.

Entretanto, o legado de uma revista não pode ser medido apenas por seu fator de impacto. Ao longo desses oito anos, procuramos preservar e fortalecer a publicação bilíngue, gratuita e de acesso aberto; modernizar o sistema de submissão e avaliação dos manuscritos; ampliar a participação de editores, revisores e autores internacionais; valorizar os programas brasileiros de pós-graduação; contextualizar os artigos originais por meio dos minieditoriais; e integrar mais intensamente o portal da revista às redes científicas e sociais.^1-5^ O objetivo comum dessas iniciativas foi aproximar a ciência de seus leitores e oferecer aos autores uma plataforma editorial rigorosa, ágil e internacionalmente visível.

A expansão da presença digital confirma essa aproximação. A média mensal de usuários nas páginas do ABC Cardiol passou de aproximadamente 34,8 mil em 2023 para 44,7 mil em 2024 e 54,8 mil entre janeiro e outubro de 2025. Somente em outubro de 2025, foram registrados 94,5 mil usuários. Esses dados demonstram que a influência de um periódico científico contemporâneo se estende muito além de seus fascículos e de suas métricas tradicionais, alcançando leitores por meio de diferentes plataformas, formatos e formas de disseminação do conhecimento.

Outra convicção que orientou nossa atuação foi a de que os periódicos científicos brasileiros não devem ser considerados uma alternativa secundária, mas o verdadeiro lar de parte substancial da melhor ciência produzida em nosso país. Por isso, defendemos o reconhecimento adequado do ABC Cardiol nos sistemas nacionais de avaliação e estimulamos a colaboração, e não a competição, entre as revistas cardiovasculares.^6-8^ A articulação da Família de Publicações Científicas da SBC e os mecanismos de transferência de manuscritos procuraram ampliar as possibilidades de publicação de estudos de qualidade, preservando-os no ecossistema científico nacional e oferecendo-lhes visibilidade internacional.

Nenhum desses resultados pertence a uma única pessoa ou a uma única gestão. Eles são fruto do trabalho de autores, revisores, editores associados, membros dos conselhos editoriais, assistentes editoriais, colaboradores, dirigentes da SBC e de todos os editores-chefes que construíram a história dos *Arquivos Brasileiros de Cardiologia*. Entre essas pessoas, destaque deve ser feito à professora Gláucia Maria Moraes de Oliveira, editora associada durante a gestão 2018-2025, parceira constante nas decisões e na defesa da ciência brasileira, e que, desde janeiro de 2026, assumiu a editoria-chefe. Sua liderança representa simultaneamente continuidade, renovação e a segurança de que o ABC Cardiol seguirá avançando.

Ao final desse período, permanece, acima de tudo, um sentimento de profunda gratidão. A revista é maior do que qualquer um de seus editores, e sua história não é composta por capítulos isolados, mas por um processo contínuo de construção. O período de 2018 a 2025 contribuiu para fortalecer sua identidade, ampliar seu impacto e aproximá-la ainda mais da comunidade científica. Mais do que o encerramento de uma etapa, a transição da editoria-chefe representa a continuidade de uma trajetória sob uma liderança que conhece profundamente a revista e que certamente a conduzirá a novos patamares. O futuro do ABC Cardiol continua sendo escrito pela comunidade científica brasileira e para a ciência cardiovascular mundial.
