# Supplementary material for: ABC Cardiol de 2018–2025: Identidade, Impacto e Construção Coletiva
Source: Arq Bras Cardiol. 2026 Sep 21;123(9):e20260582. [Article in Portuguese] doi: 10.36660/abc.20260582 (PMC13649063; doi:10.36660/abc.20260582)
Supplement: Translation

# Translation: ABC Cardiol 2018–2025: Identity, Impact, and Collective Achievement

**DOI:** 10.36660/abc.20260582i

**Authors:** Carlos Eduardo Rochitte

**Affiliations:** 1 Brazil Instituto do Coração do Hospital das Clínicas da Faculdade de Medicina da Universidade de São Paulo, São Paulo, SP – Brazil; 2 Brazil Hospital do Coração (Hcor), Associação Beneficente Síria, São Paulo, SP – Brazil; 3 Brazil Alta/DASA Medicina Diagnóstica, São Paulo, SP – Brazil; 4 Brazil Blume Medicina Diagnóstica, São Paulo, SP – Brazil

**Keywords:** Periodical, Journal Impact Factor, Scientific and Technical Publications, Scientific Publication Indicators

The period beginning in 2018 marked a new stage in the editorial trajectory of the *Arquivos Brasileiros de Cardiologia*. At that time, two pillars were established as priorities for the journal’s development: expanding its internationalization and increasing its scientific impact.^1^ The impact factor remained just above 1, and the announcement of an impact factor of 1.318 was therefore celebrated as an important achievement for the Brazilian Society of Cardiology and the entire cardiovascular scientific community.^2^ Beyond achieving numerical targets, the goal was to strengthen the journal’s ability to disseminate cardiovascular science produced in Brazil and make it increasingly visible, relevant, and accessible internationally.

One of the symbolic changes during this period was the consolidation of the name ABC Cardiol. The journal’s official and historic name, *Arquivos Brasileiros de Cardiologia*, was fully preserved, while the journal also began to be presented under a shorter name that was easily recognizable and better able to convey its identity within the international scientific community. Over time, ABC Cardiol became more than an abbreviation; it developed into an editorial brand widely embraced by authors, readers, reviewers, and the Brazilian Society of Cardiology itself.

The numbers reflect part of this trajectory. The impact factor increased from 1.318 for 2017 to 1.679 the following year and reached 2.0 in 2020.^2-4^ It subsequently achieved an all-time high of 2.667, more than twice the level recorded at the beginning of the 2018–2025 editorial term. Following a transient decline to 1.9, the most recent impact factor has risen again to 2.3 (Figure 1). Although naturally subject to the fluctuations inherent to bibliometric indicators, this evolution demonstrates the journal’s consolidation at a new level of visibility and, above all, its capacity for recovery and sustained scientific influence.

**Figure 1:** – Impact Factor of the Arquivos Brasileiros de Cardiologia – Temporal Evolution.

However, the legacy of a scientific journal cannot be measured by its impact factor alone. Over these eight years, efforts were made to preserve and strengthen bilingual, free, open-access publication; modernize the manuscript submission and evaluation system; expand the participation of international editors, reviewers, and authors; support Brazilian graduate programs; place original articles into context through invited mini-editorials; and more fully integrate the journal’s website with scientific and social media platforms.^1-5^ The common goal of these initiatives was to bring science closer to its readers and provide authors with a rigorous, efficient, and internationally visible editorial platform.

The expansion of the journal’s digital presence further illustrates this closer connection with its readership. The average monthly number of users accessing ABC Cardiol pages increased from approximately 34,800 in 2023 to 44,700 in 2024 and 54,800 between January and October 2025. In October 2025 alone, 94,500 users were recorded. These figures demonstrate that the influence of a contemporary scientific journal extends far beyond its issues and traditional metrics, reaching readers through multiple platforms, formats, and approaches to the dissemination of knowledge.

Another conviction that guided this work was that Brazilian scientific journals should not be regarded as a secondary alternative, but rather as the true home for a substantial share of the best science produced in our country. Accordingly, appropriate recognition of ABC Cardiol within national evaluation systems was advocated, while collaboration rather than competition among cardiovascular journals was encouraged.^6-8^ The development of the SBC Family of Scientific Publications and the implementation of manuscript transfer mechanisms sought to broaden publication opportunities for high-quality studies, retaining them within the national scientific ecosystem while providing international visibility.

None of these achievements belongs to a single individual or a single editorial term. They are the result of the collective work of authors, reviewers, associate editors, editorial board members, editorial assistants, collaborators, SBC leaders, and all the editors-in-chief who have contributed to the history of the *Arquivos Brasileiros de Cardiologia*. Among them, special recognition should be given to Professor Gláucia Maria Moraes de Oliveira, who served as an associate editor during the 2018–2025 term, was a constant partner in editorial decisions and in the defense of Brazilian science, and assumed the role of editor-in-chief in January 2026. Her leadership represents continuity and renewal, while providing confidence that ABC Cardiol will continue to move forward.

At the conclusion of this period, above all, there remains a profound sense of gratitude. The journal is greater than any one of its editors, and its history is not composed of isolated chapters, but rather of a continuous process of collective construction. The period from 2018 to 2025 contributed to strengthening its identity, expanding its impact, and bringing it even closer to the scientific community. More than the conclusion of one stage, the transition in editorial leadership represents the continuation of a trajectory under the guidance of a leader who knows the journal deeply and will undoubtedly take it to new heights. The future of ABC Cardiol continues to be written by the Brazilian scientific community, for the global cardiovascular scientific community.
